# *Chrysanthemum zawadskii* ethanol extract inhibits the replication of alpha-coronavirus and beta-coronavirus

**DOI:** 10.1371/journal.pone.0326225

**Published:** 2025-06-18

**Authors:** Chansoo Kim, Jang Hoon Kim, Siyun Lee, Chunghyeon Lee, Gwi Yeong Jang, Jihun Choi, Woochul Jung, Jayhyun Park, Junsoo Park

**Affiliations:** 1 Division of Biological Science and Technology, Yonsei University, Wonju, Republic of Korea; 2 Department of Herbal Crop Research, National Institute of Horticultural & Herbal Science, RDA, Eumsung, Republic of Korea; 3 R&D team, Korea Mine Rehabilitation and Mineral Resources Corporation, Wonju, Korea; University of Alabama at Birmingham, UNITED STATES OF AMERICA

## Abstract

Coronavirus can induce various diseases, from mild common cold to severe COVID-19. Coronavirus can be continuously prevalent, similar to the influenza virus, due to its frequent mutation of the RNA genome. Therefore, diverse methods are required to treat coronavirus-related diseases. *Chrysanthemum zawadskii*, found in Asian countries including Korea, has been traditionally used to treat various diseases, including cough, pneumonia, and common cold. However, the antiviral effects of *Chrysanthemum zawadskii* have not been reported yet. Here, we demonstrated that *Chrysanthemum zawadskii* ethanol extract (CZE) treatment interferes with the replication of alpha-coronavirus (HCoV-229E) and beta-coronavirus (HCoV-OC43). CZE treatment ameliorates beta-coronavirus-induced cytotoxicity and reduces plaque formation. CZE treatment inhibits the coronavirus protein and RNA expression, indicating CZE inhibits coronavirus replication. HPLC analysis showed that CZE contains many antiviral compounds, such as luteolin and isochlorogenic acid. These results indicate that CZE is a potent medicinal herb for treating coronavirus-related diseases.

## Introduction

Coronavirus can induce various diseases, from mild common cold to severe COVID-19 [1]. Common cold is caused by the infection of various viruses such as rhinovirus and adenovirus, and a significant portion of the common cold are caused by coronavirus [2,3]. Coronaviruses are a group of enveloped, positive-sense single stranded RNA viruses belonging to the family *Coronaviridae* [4]. Coronavirus can be persistently prevalent, similar to influenza virus, due to its frequent mutation of the RNA genome [5]. The occurrence of drug- or vaccine-resistant coronavirus can cause serious problems. For example, the omicron variant exhibits significant immune evasion, reducing the effectiveness of existing mRNA vaccines [6]. Additionally, certain variants were also resistant to monoclonal antibody treatments such as bamlanivimab [7]. Therefore, diverse methods are required to treat the coronavirus-related diseases.

Coronaviruses infect a wide range of hosts, including mammals and birds, and are classified into alpha, beta, gamma, and delta coronavirus. Only alpha and beta coronaviruses are known to infect humans [8]. Coronaviruses are important pathogens because they can spread from animals to humans and cause large outbreaks. Four human coronavirus strains (HCoV-OC43, HCoV-229E, HCoV-NL63, and HCoV-HKU1) are well known to induce the common cold, and they are responsible for 15–30% of the common cold in adults [9]. Beta coronavirus contains highly virulent coronavirus (SARS-CoV, MERS-CoV and SARS-CoV-2) [9]. Because HCoV-OC43 is a member of beta coronavirus, HCoV-OC43 is commonly used as an avirulent model for other virulent coronaviruses [10,11]. A recent study showed that the antibodies raised by seasonal human coronavirus, including HCoV-OC43, protect humans from critical COVID-19, indicating these two viruses share conserved antigenicity [12].

*Chrysanthemum zawadskii* (*C. zawadskii*) belongs to the family Asteraceae [13]. *C. zawadskii* is a perennial herb found in Asian countries including Korea (known as ‘Gu-Jeol-Cho’ in Korean) [14]. *C. zawadskii* has been traditionally used to treat various diseases such as cough, pneumonia, and common cold [15–17]. Recent studies showed that *C. zawadskii* extract treatment has an anti-inflammatory effect, and the detailed mechanisms were provided [18–21]. *C. zawadskii* extract treatment was also reported to alleviate the bone- and muscle-related diseases such as osteoarthritis and muscle atrophy [22–30]. In addition, recent studies showed that *C. zawadskii* treatment is helpful to hair growth [15,31,32]. However, the antiviral effect of *C. zawadskii* treatment has not been reported yet.

A significant portion of common cold cases are associated with human coronavirus infection, and *C. zawadskii* was traditionally used to treat common cold, pneumonia, and cough [15]. Here, we report that *C. zawadskii* ethanol extract (CZE) treatment interferes with the replication of beta-coronavirus (HCoV-OC43) and alpha-coronavirus (HCoV-229E). We also demonstrated that CZE ameliorates beta-coronavirus-induced cytotoxicity.

## Materials and methods

### Material and sample preparation

The aerial parts of CZE were collected and dried in August 2021 at Chungcheongbuk province, Republic of Korea, and were identified by Dr. J.H. Kim in the Department of Herbal Crop Research (DHCR), National Institute of Horticultural and Herbal Science (NIHHS). A sample specimen (CZA2108) was deposited at the Herbarium of DHCR, NIHHS. This plant’s dried aerial parts (10 g) were extracted three times with ethanol (40 mL). The weight of the concentrated solution after filtration was 750 mg. The sample for HPLC analysis was prepared by dissolving 2 mg of extract in 1 mL of 70% ethanol and then filtering with a membrane filter (0.2 µm, Pall Co., Ann Arbor, Mi, USA).

### HPLC analysis

The sample was analyzed using an Agilent 1200 HPLC system (Agilent Technologies, Santa Clara, CA, USA). The mobile phase consisted of 0.1% formic acid in ACN (A) in acetonitrile and 0.1% formic acid in water, using the following: 0 min (2% A), 0−5 min (2−2% A), 5−12 min (2−5% A), 12−17 min (5−8% A), 17−65 min (8−30% A), 65−68 min (30−30% A), 68−78 min (30−50% A), 78−100 min (50−100% A), and 100−110 min (100−100% A). The injection volume, flow rate, and detection wave length were set at 10 μL, 1.0 mL/min, and 340 nm, respectively. The column was YMC-triart (3 μm, 100 × 4.6 mm, YMC Co., Kyoto, Japan).

### Infection of coronavirus

HCoV-OC43 virus was purchased from ATCC (Rockville, MD, USA) and rhabdomyosarcoma (RD) cells were obtained from Korean Cell Line Bank (Seoul, Korea). RD cells were maintained in DMEM media (Welgene, Seoul, Korea) supplemented with 10% FBS (Thermo Fisher Scientific, Waltham, MA, USA). RD cells were infected with human coronavirus (10^6^ PFU/ml), as described previously [10]. Briefly, cells were incubated with the indicated dilutions of media containing coronavirus (MOI of 0.01), and the infected cells were maintained in MEM media supplemented with 2% FBS. For plaque formation, RD cells were incubated in a 12-well plate and infected with the indicated concentration of media containing coronavirus. Later, the infected cells were covered with the overlay medium (0.3% agarose). The infected cells were incubated for an additional 4 days and stained with 0.1% crystal violet solutions. Cell viability was measured using MTT assay as described previously [33]. All experiments using RD cells and HCoV-OC43 virus were conducted in accordance with approved biosafety protocols (BSL-2 level) (Supporting information #1-#4).

### Western blot and immunofluorescent imaging

Western blot with anti-HCoV-OC43 antibody was used to evaluate the expression level of coronavirus proteins. Cells and conditioned media were separately collected and prepared with cell lysis buffer (50 mM NaCl, 50 mM HEPES (pH 7.5), and 1% NP40) containing a protease inhibitor. Prepared lysates were separated with SDS-PAGE and blotted into PVDF membrane filters (Bio-Rad, Hercules, CA, USA). Coronavirus proteins were detected with an anti-HCoV-OC43 antibody (Sigma-Aldrich, Saint Louis, MO, USA), and the membrane was blocked with 3% bovine serum albumin in TBS containing 0.1% Tween 20. Confocal analysis of the fluorescence was performed using a Zeiss LSM 800 confocal microscope (Carl Zeiss, Oberkochen, Germany) at the Neuroscience Translational Research Solution Center (Busan, South Korea).

### Quantitative RT-PCR

Quantitative RT-PCR was used to measure the level of coronavirus RNA in the cells and conditioned media. Cells and media were separately collected, and total RNA was prepared using TRIZOL (Thermo Fisher Scientific) as manufacturer’s protocol. An equal amount of total RNA was used to synthesize cDNA using the M-MLV cDNA Synthesis kit (Enzynomics, Seoul, Korea). StepOnePlus Real-Time PCR system (Thermo Fisher Scientific) was used for qRT-PCR, and GAPDH gene was used as a control. Detailed primer sequences were described previously [10].

### Scanning electron microscope (SEM)

For SEM imaging, RD cells were grown on sterilized 9 mm coverslips and infected with HCoV-OC43 for 3 days. Cells were fixed with 2.5% glutaraldehyde for 1 h and dehydrated in an ethanol series (20%, 40%, 60%, 80%, 90% and 100% Et-OH). After dehydration, the cells were air-dried using a vacuum desiccator for 1 h. Cells were coated with platinum, and images were captured using a Carl Zeiss SEM SUPRA 40 microscope (Carl Zeiss).

### Statistical analysis

The results of Western blot, quantitative RT-PCR and MTT were evaluated by a 2-tailed Student’s t-test using Excel software (Microsoft, Redmond, WA, USA). A p-value of 0.05 was considered significant.

## Results

### *Chrysanthemum zawadskii* ethanol extract (CZE) decreased the expression of alpha coronavirus and beta coronavirus proteins

**Fig 1:**
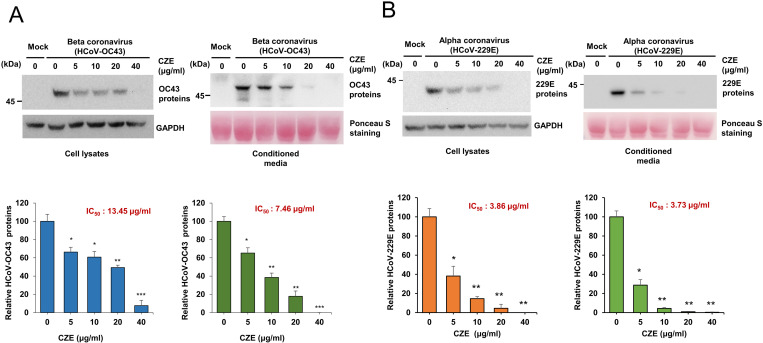
*Chrysanthemum zawadskii* ethanol extract (CZE) decreases the expression of coronavirus protein. (A) RD cells were infected with human beta-coronavirus (HCoV-OC43) and treated with the indicated concentration of CZE. After 72 h of incubation, cell lysates and conditioned media were collected, and the amount of coronavirus protein was evaluated by Western blot (Top panel). Western bands were quantified and shown in the graph (bottom panel). (B) RD cells were infected with human alpha-coronavirus (HCoV-229E) and treated with the indicated concentration of CZE. Cell lysates and conditioned media were collected and subjected to Western blot (Top panel). The expression level of HCoV-229E proteins was quantified and shown in the graph (Bottom panel). Results are shown as mean ± SEM. Panel A includes five independent replicates (n = 5) for both lysate and media. Panel B includes four repeats for lysate and three replicates (n = 3) for media. Mock vs. CZE treatment: * p < 0.05, ** p < 0.01, *** p < 0.001.

We attempted to find the plant extracts that inhibit coronavirus replication by examining the level of coronavirus. We found that *Chrysanthemum zawadskii* ethanol extract (CZE) decreases the expression of coronavirus protein. We evaluated the expression level of coronavirus proteins in the cells and conditioned media. Human beta-coronavirus infection (HCoV-OC43) results in the expression of coronavirus proteins, and CZE treatment decreased the expression level of coronavirus significantly (Fig 1A, top panel). We also quantitated the expression level of coronavirus proteins, and IC_50_ in the conditioned media was lower than IC_50_ in cell lysates (Fig 1A, bottom panel). These results indicate that CZE treatment decreased the expression of human beta-coronavirus (HCoV-OC43).

Because CZE treatment decreased beta-coronavirus (HCoV-OC43) replication, we also examined whether CZE can decrease alpha-coronavirus replication. We used HCoV-229E strain to test whether CZE can inhibit alpha-coronavirus replication. CZE treatment decreased the expression level of HCoV-229E in cells and the conditioned media (Fig 1B). Moreover, the IC_50_ of CZE against HCoV-229E was lower than that against HCoV-OC43, suggesting greater sensitivity of HCoV-229E to CZE treatment (Fig 1B). These results indicate that CZE is effective against both strains.

### *Chrysanthemum zawadskii* ethanol extract (CZE) ameliorates beta-coronavirus-induced cytotoxicity

**Fig 2:**
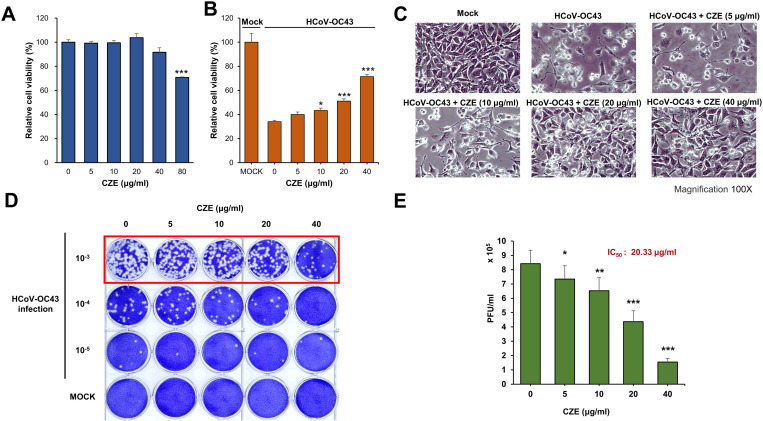
*Chrysanthemum zawadskii* ethanol extract (CZE) inhibits coronavirus induced cytotoxicity. (A) Cell viability was evaluated upon CZE treatment. RD cells were incubated with the indicated concentration of CZE for 24 h, and cell viability was measured using an MTT assay. (B) CZE treatment ameliorates coronavirus induced cell death. RD cells were infected with mock or coronavirus (HCoV-OC43) and treated with CZE for 72 h. (C) Microscopic images of coronavirus-infected cells with CZE treatment. (D) CZE treatment reduced coronavirus-mediated plaque formation. (E) The numbers of plaques were counted and shown in the graph. Panel A and B were performed with four independent replicates (n = 4), and results are shown as mean ± SEM. Panel E includes five independent experiments (n = 5). Mock vs. CZE treatment: * p < 0.05, ** p < 0.01, *** p < 0.001.

Because CZE treatment inhibits the expression of coronavirus proteins in the cells and conditioned media, we examined whether CZE ameliorates the coronavirus-induced cytotoxicity. Human alpha coronavirus (HCoV-229E) infection does not show the cytopathic effect, and we used human beta coronavirus (HCoV-OC43) to examine the antiviral effect of CZE [10]. We performed the cell viability assay to determine the adequate concentration to examine the antiviral effects in vitro. We found that CZE showed minimal effects up to 60 µM. However, the CZE treatment at 80 µM showed a significant decrease in cell viability (Fig 2A). Infection with beta-coronavirus (HCoV-OC43) decreases cell viability significantly, and we examined whether CZE treatment ameliorates coronavirus-induced cytotoxicity. RD cells were infected with coronavirus, treated with CZE together, and the cell viability was examined. Human beta-coronavirus (HCoV-OC43) infection results in a decrease in cell viability by up to 60%, and CZE treatment increases the viable cells in a dose-dependent manner (Fig 2B). Microscopic images showed that the number of viable cells was increased by CZE treatment (Fig 2C). These results indicate that CZE interferes with beta-coronavirus-induced cytotoxicity.

We examined the antiviral effect of CZE treatment by examining plaque formation. When RD cells were infected with human beta-coronavirus (HCoV-OC43), we observed an increase in plaque number, which was dependent on the virus titer (Fig 2D). The treatment of CZE decreased the number of coronavirus-induced plaques in a dose-dependent manner (Fig 2D, E). These results indicate that CZE treatment ameliorates coronavirus-induced cytotoxicity.

### CZE treatment decreased the replication of coronavirus

**Fig 3:**
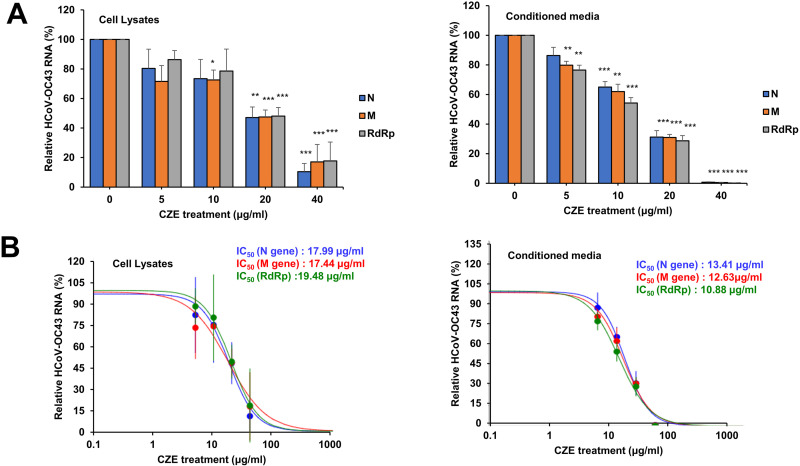
CZE treatment inhibits human coronavirus replication. (A) RD cells were infected with coronavirus (HCoV-OC43) and treated with CZE for 72 h. Total RNA was isolated from the cells and conditioned media, and the level of each RNA was evaluated using quantitative RT-PCR. (B) The IC_50_ of CZE was determined and shown in the graph. Panel A includes five independent replicates (n = 5) for both cell lysates and conditioned media, and results are shown as mean ± SEM. Mock vs. CZE treatment: * p < 0.05, ** p < 0.01, *** p < 0.001.

Coronavirus contains RNA genomes, and we evaluated the level of coronavirus RNA upon CZE treatment. We evaluated the coronavirus RNA level using quantitative RT-PCR and used the region of nucleoprotein (N), membrane protein (M), and RNA-dependent RNA polymerase. CZE treatment decreased the RNA level of human coronavirus (HCoV-OC43) in a dose-dependent manner in cells and conditioned media (Fig 3A). We also determined IC_50_ and IC_50_ in the conditioned media are lower than IC_50_ in the cells (Fig 3B). The coronavirus RNA in the conditioned media is mainly derived from the released virus particle, and these results indicate that CZE treatment inhibits coronavirus replication.

### CZE treatment decreases the number of infected cells and virus particle production

**Fig 4:**
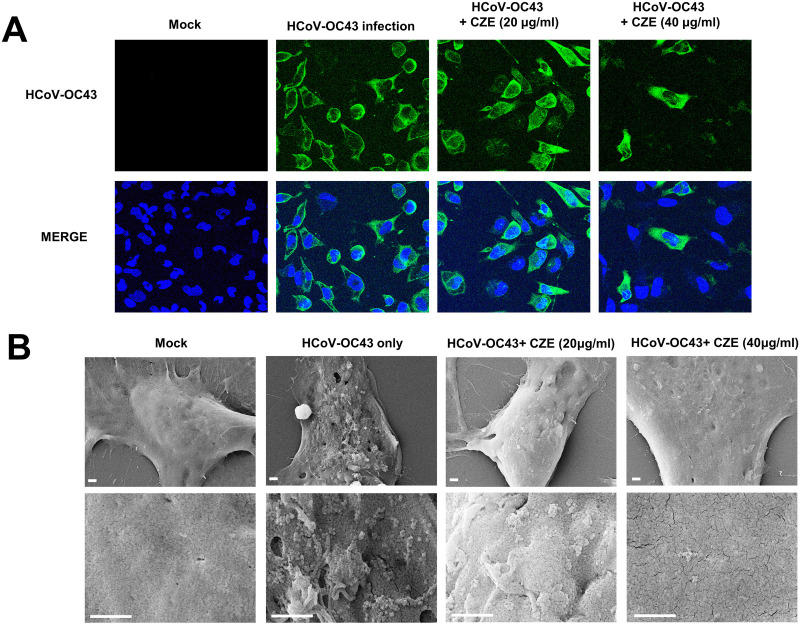
CZE treatment inhibits the coronavirus production. (A) CZE treatment reduces the number of coronavirus-infected cells. RD cells were infected with the coronavirus (HCoV-OC43) and treated with CZE. The infected cells were fixed and immunostained with anti-HCoV-OC43 antibody. (B) CZE treatment inhibits the coronavirus production. Cells were analyzed by a scanning electron microscope (SEM). Scale Bars 1 μm.

Because CZE treatment decreased coronavirus proteins and RNA, we examined the number of infected cells by CZE treatment. RD cells were infected with beta coronavirus (HCoV-OC43) and immune-stained with anti-OC43 antibody. When the cells were treated with CZE, we observed that the number of infected cells decreased (Fig 4A). These results indicate that CZE treatment decreased the infected cells.

Next, we examined the coronavirus infection on the cell surface. RD cells were infected with HCoV-OC43, and we used a scanning electron microscope (SEM) to analyze the infected cell surface. HCoV-OC43 infected cells have surface projections and virus particles on the infected cell surface as described previously [34]. CZE treatment decreased coronavirus-induced virus particle production and surface projections in a dose-dependent manner (Fig 4B). These results indicate that CZE treatment decreased the coronavirus replication and infectivity.

### HPLC analysis of CZE

**Fig 5:**
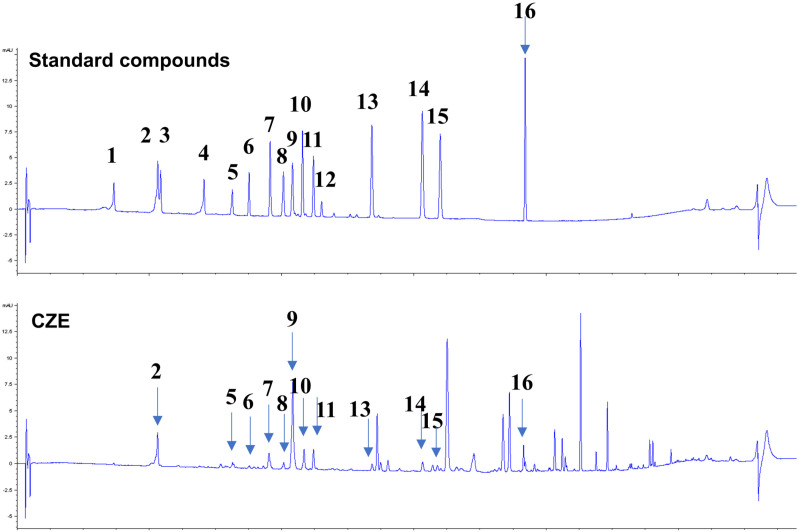
Antiviral compounds of CZE were determined by HPLC analysis. The single compounds of CZE were analyzed by HPLC and their numbers are indicated in the graph. (**1**, neochlorogenic acid; **2**, chlorogenic acid; **3**, 4-*O*-caffeoylquinic acid; **4**, 1,3-di-*O*-caffeoylquinic acid; **5**, vicenin; **6**, isochaftoside; **7**, luteolin-7-*O*-glucoside; **8**, isochlorogenic acid B; **9**, isochlorogenic acid A; **10**, apigenin-7-*O*-glucoside; **11**, isochlorogenic acid; **12**, biosmetin-7-*O*-glucoside; **13**, luteolin; **14**, apigenin; **15**, diosmetin; **16**, acacetin).

We showed that the CZE extract interfered with human coronavirus replication, and we attempted to examine whether CZE contains the antiviral single compounds. HPLC analysis was used to find an active single compound in CZE and the respective signals of CZE, and a mixture of standard compounds were analyzed by HPLC [35]. They were detected at 340 nm for 110 min and each peak of CZE was identified by comparing those with the retention times of the standard compounds. Twelve compounds were detected in the CZE (2: chlorogenic acid, 21.2 min; 5: vicenin, 32.5 min; 6: isochaftoside, 35.0 min; 7: luteolin-7-*O*-glucoside, 38.2 min; 8: isochlorogenic acid B, 40.2 min; 9: isochlorogenic acid A, 41.6 min; 10: apigenin-7-*O*-glucoside, 43.1 min; 11: isochlorogenic acid, 44.7 min; 13: luteolin, 53.6 min; 14: apigenin, 61.2 min; 15: diosmetin, 63.9 min; 16: acacetin, 76.7 min) (Fig 5).

## Discussion

In this report, we attempted to find an herbal plant that is effective in treating coronavirus-related diseases. *Chrysanthemum zawadskii* was traditionally used to treat cough, common cold, and pneumonia, and coronavirus infection causes similar diseases, including common cold and pneumonia. A recent study showed that 15–30% of common cold is related with coronavirus infection [2]. Therefore, the inhibition of coronavirus replication by *C. zawadskii* can be related to treating of common cold and pneumonia.

When we examined the effect of *C. zawadskii* on coronavirus replication, we examined the effect of CZE on cell viability. CZE treatment did not show the significant cytotoxicity up to 40 µg/mL (Fig 2A). However, CZE can significantly inhibit the coronavirus protein and RNA expression at 5 µg/mL (Fig 1). Therefore, CZE can be treated without significant cytotoxicity, when the concentration of CZE is used below 40 µg/mL.

When we analyzed CZE using HPLC, we found many phenolic compounds in CZE. We found that CZE contains luteolin, apigenin, chlorogenic acid, and isochlorogenic acid. They are reported to have the potential to inhibit the replication SARS-CoV-2 *in silico*. Chlorogenic acid and isochlorogenic acid were reported to be a potent inhibitor of coronavirus replication [36–38]. *In silico* screening study showed that luteolin and apigenin are potent protease inhibitors of coronavirus [39,40]. These results indicate that CZE contains many potent antiviral compounds.

Here, we demonstrated that *C. zawadskii* extract (CZE) inhibits the replication of human alpha coronavirus (HCoV-229E) and human beta coronavirus (HCoV-OC43). Moreover, CZE ameliorates coronavirus-induced cytotoxicity. HPLC analysis showed that CZE contains many potent antiviral compounds. Because the ethnopharmacological evidence and the biological experiment data support CZE’s potential for treating coronavirus, CZE can be considered a candidate for this treatment. Further animal experiments should be followed to confirm its pharmacological effect on coronavirus-related diseases.

## Supporting information

S1 Fig Uncropped western blot images corresponding to Fig 1. (PDF)

S2 Fig The raw MTT data for Fig 2A and Fig 2B. (CSV)

S3 Fig Plaque formation data corresponding to Fig 2D. (CSV)

S4 Fig The quantitative RT-PCR data for Fig 3. (CSV)
