# Supplementary material for: Chrysanthemum zawadskii ethanol extract inhibits the replication of alpha-coronavirus and beta-coronavirus
Source: PLoS One. 2025 Jun 18;20(6):e0326225. doi: 10.1371/journal.pone.0326225 (PMC12176134; doi:10.1371/journal.pone.0326225)
Supplement: S1 Fig — (PDF) [file pone.0326225.s001.pdf]

# Fig 1A

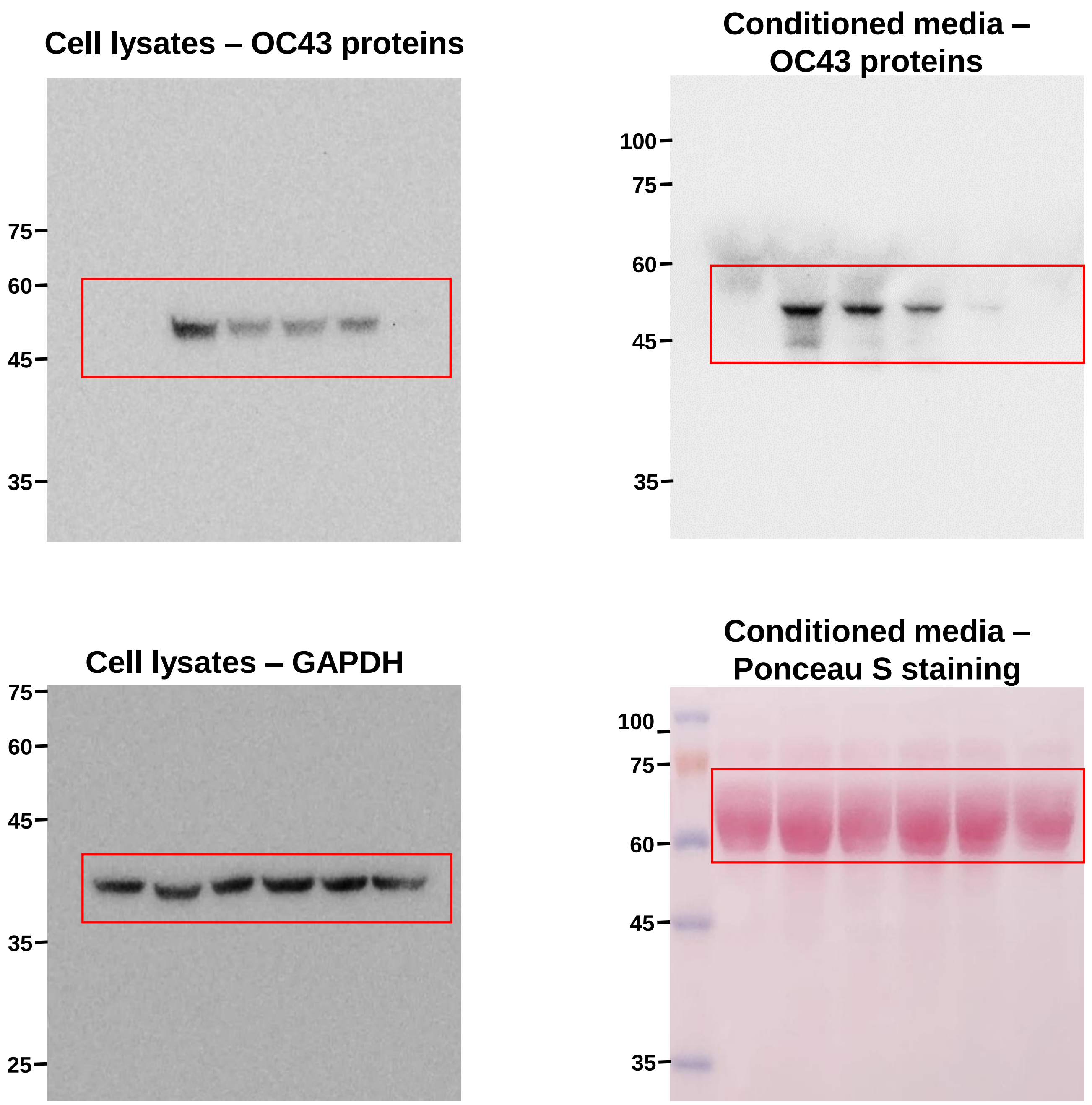
Cell lysates – OC43 proteins
75
60
45
35
Conditioned media – OC43 proteins
100
75
60
45
35
Cell lysates – GAPDH
75
60
45
35
25
Conditioned media – Ponceau S staining
100
75
60
45
35

## Fig 1B

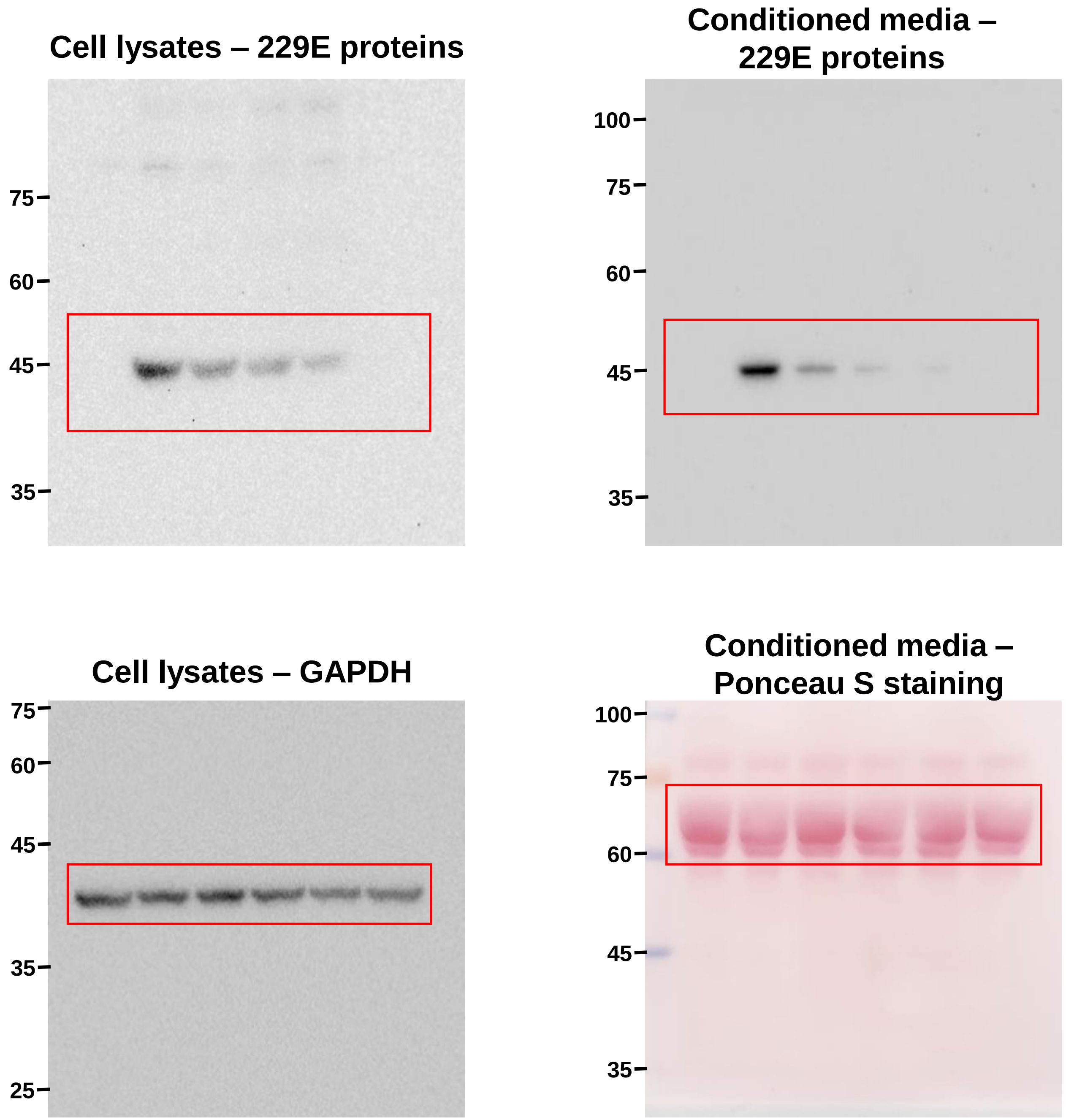
Cell lysates – 229E proteins
75
60
45
35
Conditioned media – 229E proteins
100
75
60
45
35
Cell lysates – GAPDH
75
60
45
35
25
Conditioned media – Ponceau S staining
100
75
60
45
35
